# Enzymatic, immunological and phylogenetic characterization of *Brucella suis *urease

**DOI:** 10.1186/1471-2180-8-121

**Published:** 2008-07-19

**Authors:** Araceli Contreras-Rodriguez, Jose Quiroz-Limon, Ana M Martins, Humberto Peralta, Eric Avila-Calderon, Nammalwar Sriranganathan, Stephen M Boyle, Ahide Lopez-Merino

**Affiliations:** 1 Escuela Nacional de Ciencias Biológicas, I.P.N. México, Prol. Carpio y Plan de Ayala s/n, Col. Sto. Tomas, CP 11340, Mexico; 2 Center for Molecular Medicine and Infectious Diseases, Virginia-Maryland Regional College of Veterinary Medicine, Virginia Tech, 1410 Prices Fork Rd., Blacksburg, VA 24061-0342, USA; 3 Virginia Bioinformatics Institute, Virginia Tech, Washington Street, Blacksburg, VA 24061-0477, USA; 4 Programa de Genómica Funcional de Procariotes, Centro de Ciencias Genómicas, Universidad Nacional Autónoma de México, Av. Universidad s/n, PO Box 565-A, Cuernavaca, Morelos, CP 62210, Mexico

## Abstract

**Background:**

The sequenced genomes of the *Brucella *spp. have two urease operons, *ure*-1 and *ure*-2, but there is evidence that only one is responsible for encoding an active urease. The present work describes the purification and the enzymatic and phylogenomic characterization of urease from *Brucella suis *strain 1330. Additionally, the urease reactivity of sera from patients diagnosed with brucellosis was examined.

**Results:**

Urease encoded by the *ure*-1 operon of *Brucella suis *strain 1330 was purified to homogeneity using ion exchange and hydrophobic interaction chromatographies. The urease was purified 51-fold with a recovery of 12% of the enzyme activity and 0.24% of the total protein. The enzyme had an isoelectric point of 5, and showed optimal activity at pH 7.0 and 28–35°C. The purified enzyme exhibited a Michaelis-Menten saturation kinetics with a *K*_*m *_of 5.60 ± 0.69 mM. Hydroxyurea and thiourea are competitive inhibitors of the enzyme with K_i _of 1.04 ± 0.31 mM and 26.12 ± 2.30 mM, respectively. Acetohydroxamic acid also inhibits the enzyme in a competitive way. The molecular weight estimated for the native enzyme was between 130–135 kDa by gel filtration chromatography and 157 ± 7 kDa using 5–10% polyacrylamide gradient non-denaturing gel. Only three subunits in SDS-PAGE were identified: two small subunits of 14,000 Da and 15,500 Da, and a major subunit of 66,000 Da. The amino terminal sequence of the purified large subunit corresponded to the predicted amino acid sequence encoded by *ureC1*. The UreC1 subunit was recognized by sera from patients with acute and chronic brucellosis. By phylogenetic and cluster structure analyses, *ureC1 *was related to the *ureC *typically present in the *Rhizobiales*; in contrast, the *ureC2 *encoded in the *ure*-2 operon is more related to distant species.

**Conclusion:**

We have for the first time purified and characterized an active urease from *B. suis*. The enzyme was characterized at the kinetic, immunological and phylogenetic levels. Our results confirm that the active urease of *B. suis *is a product of *ure*-1 operon.

## Background

*Brucella *spp. causes brucellosis, a zoonotic disease still endemic in many countries of the world. This infectious disease affects different animal species and is transmitted to humans in several ways, the most common through ingestion of raw milk or other unpasteurized dairy products. The preferred ecological niche for the brucellae is within phagosomal compartments of host macrophages; the capacity of this bacterial pathogen to establish and maintain chronic infections is dependent upon its ability to replicate within these phagocytic cells [1]. *Brucella *belongs to the alpha-2 subdivision of the *Proteobacteria *and they are therefore phylogenetically related to the plant cell-associated species of the genera *Rhizobium *and *Agrobacterium *[2].

A wide variety of environmentally and medically important bacteria produce the enzyme urease (urea amidohydrolase; EC 3.5.1.5), which catalyzes the hydrolysis of urea, leading to the production of carbon dioxide and ammonia [3]. This enzyme allows many soil bacteria to use urea as a nitrogen source. Urease is also an important virulence factor that improves survival of pathogenic bacteria under acidic conditions within the host and can also cause direct damage to the host tissue due to ammonia, CO_2 _or alkali production [4,5]. Interestingly, some species of *Rhizobiales*, such as *Brucellae *and *Bradyrhizobium *BTAi1 show multiple urease clusters [6]. This reiteration so far is found in other bacterial species, namely *Streptomyces coelicolor*, *S. avermitilis, Pseudomonas syringae *and *Escherichia coli *[7].

Several bacterial ureases have already been purified and characterized [5]. One of the most studied ureases is from *Helicobacter pylori*, a bacterium that is able to persist in the stomach where the pH is very acidic [8]. Urease activity is an important colonization factor by generating ammonia in the immediate bacterial microenvironment, thus protecting *H. pylori *from the deleterious effects of gastric acid [9]. Furthermore, urease activity appears to be responsible for the acid resistance of the invasive enteric pathogen *Yersinia enterocolitica *[10]. Interestingly, some humans have a genetic predisposition to develop reactive arthritis following a *Y. enterocolitica *infection, which correlates to their serum reactivity with the UreB subunit of *Yersinia *urease [11].

The present work describes the purification as well as the enzymatic and phylogenomic characterization of urease from *Brucella suis *strain 1330; in addition, the urease reactivity of sera from patients diagnosed with brucellosis was examined. To our knowledge, no *Brucella *urease has been previously purified and characterized. Since *Brucella *is a human pathogen, it is important to obtain as much information as possible to complement databases, such as BRENDA [12-14]. These results can then be used by experimentalists and modelers to understand, from a systems biology point of view [15], the mode of action of enzymes in the pathogen and how that can be modified in order to develop new disease treatments.

## Results and discussion

**Figure 1:**
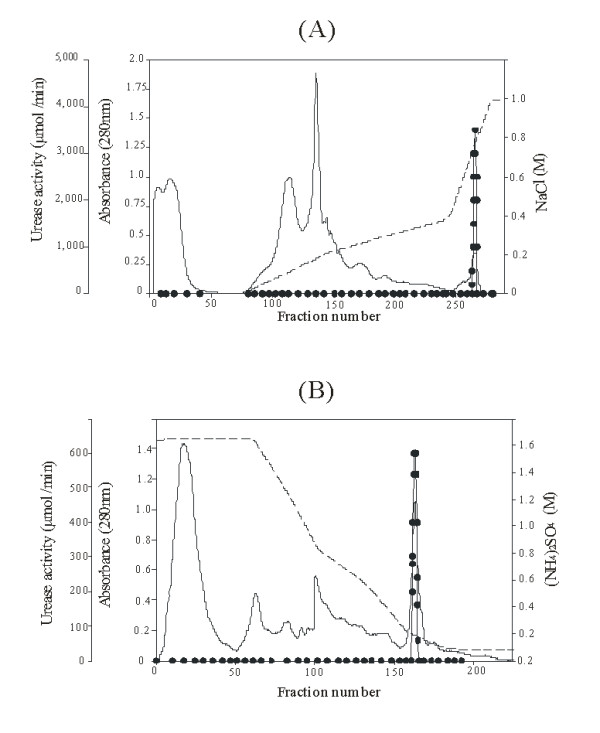
**High Performance Q Sepharose and Source PHE chromatography of *Brucella suis *urease**. Column elautes were monitored for absorbance 280 nm (solid lines), and fractions were assayed for urease activity (solid circles), as described in Methods. A) High Performance Q Sepharose chromatography was performed using a NaCl gradient (dotted line); fractions with urease activity where precipitated with (NH_4_)_2_SO_4 _(1.5 M) and applied to the Source PHE column. B) Source PHE chromatography was performed using a (NH_4_)_2_SO_4 _gradient (dotted line). Urease was eluted in 180 mM of (NH_4_)_2_SO_4_.

Urease was purified from the soluble extract of *B. suis *1330 using two consecutive chromatography steps that resulted in the final isolation of a homogeneous enzyme (Figure 1). As previously reported in the case of *Mycobacterium tuberculosis *[16], hydrophobic-interaction chromatography was consistently the most useful method to purify *B. suis *urease. The urease was purified 51-fold, with a recovery of 12% of total urease activity and 0.24% of the total protein (Table 1).

**Table 1:** Purification of *Brucella suis *1330 urease.

Purification step	Protein (mg)	Total activity (μmol/min)	Enzyme recovery (%)	Specific activity (μmol urea/min per mg of protein)	Purification (fold)
Crude extract	498	5229	100	10.5	1
Q sepharose	31	3472	66	112	10.6
Source PHE	1.2	648	12	540	51.4

Purification data refers to a single preparation, obtained in a representative experiment. The enzyme was purified by ion exchange and hydrophobic interaction chromatographies. Enzyme activity was determined using a spectrophotometric coupled assay with glutamate dehydrogenase. Details are provided in Methods section.

Urease activity was always eluted as a single symmetrical peak at 450 mM NaCl during anion exchange chromatography, and eluted at 180 mM of NH_4_SO_4 _in hydrophobic interaction chromatography. During the chromatographic purification, all fractions were measured for urease activity and consistently produced one activity at the same salt concentrations. The purified protein showed a specific activity of 540 U/mg. This is in the range of urease specific activities described for other organisms: from very low – 8 U/mg for *Rhodobacter capsulatus *[17] – to as high as 3500 U/mg in *Brevibacterium ammoniagenes *[18].

**Figure 2:**
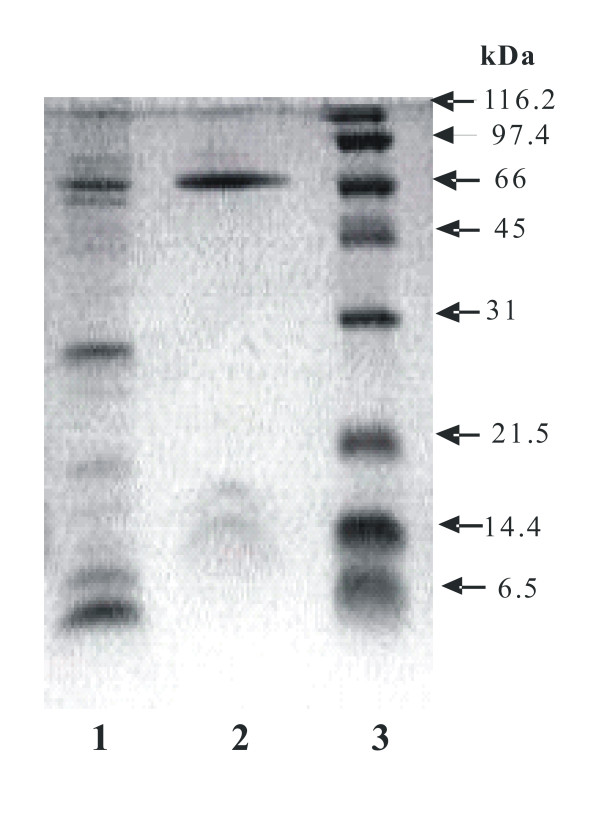
**Sodium dodecyl sulfate-polyacrylamide gel electrophoresis (15%) of *Brucella suis *urease after two steps of purification**. Lanes: 1, High Performance Q-sepharose (Ion-Exchange Chromatography), 5 μg of protein; 2, Source PHE (Hydrophobic Interaction Chromatography), 7 μg of protein; 3, standard molecular mass markers (size are indicated). Gel was stained with Coomasie blue.

**Figure 3:**
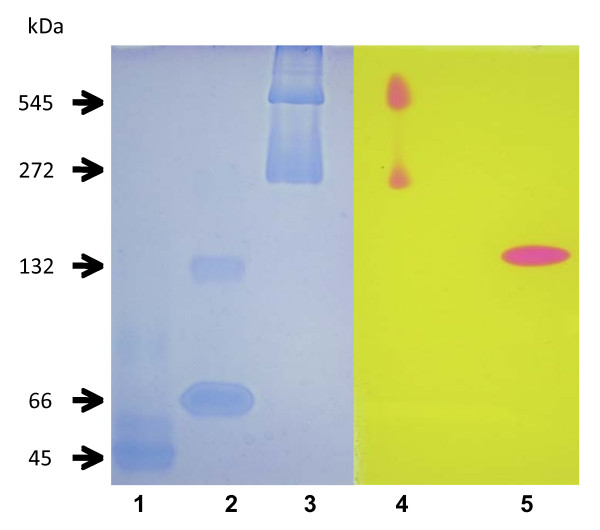
**5–10% polyacrylamide gradient nondenaturing gel electroforesis**. Lane 1, albumin from chicken egg white, 45,000 Da; lane 2, albumin from bovine serum, 66,000 Da (monomer) and 132,000 Da (dimer); lane 3, jack bean urease, 272,000 Da (trimer), and 545,000 Da (hexamer). Lanes 1–3 were stained with Coomassie blue. Lane 4, jack bean urease; lane 5, *Brucella suis *urease (157,000 Da ± 7010). Determination of urease activitiy was performed as described in Methods section. Four different gels were done to calculate the molecular weight of *Brucella suis *urease.

An SDS-PAGE analysis of the purified enzyme revealed the presence of three protein bands with apparent molecular masses of 66,000, 15,500 and 14,000 Da; the low molecular weight bands were poorly stained in contrast to the large subunit (Figure 2). The stoichiometry of the subunits in bacterial ureases has been the subject of considerable investigation. Separation of individual subunits by SDS-PAGE and integration of the staining intensities for the multiple bands reveal near-integer values for the subunits stoichiometries, but these ratios vary for the enzymes from different sources. It is likely that all ureases possess equal numbers of each or their distinct subunits polypeptides [5]. The molecular weight of the native enzyme obtained by gel filtration on Superose was 130–135 kDa, while molecular weight obtained by nondenaturing zymograms gels, appeared as one prominent band with a molecular weight of 157 ± 7 kDa (Figure 3). Bacterial native ureases show a range of molecular weights from 140 kDa in *S. salivarius *[19] to 300 – 600 kDa in *H. pylori *[5]. The isoelectric point was 5.0, very similar to the 5.1 value of urease from *Proteus penneri *[20]. The predicted molecular weight for the three subunits of *B. suis *urease operon 1: URE-A1 (11,129 Da), URE-B1 (11,377 Da) and URE-C1 (61,054 Da), the total molecular weight expected is 83,520 Da, this result is very closed with the molecular weight obtained in the polyacrylamide gradient non-denaturing gel if *B. suis *urease could be in a dimer form (2). Molecular weights higher than expected for the three subunits in the denaturing polyacrylmide gel could be a product of aberrant running. Differences between results in gel filtration chromatography compared with polyacrylamide gradient non-denaturing gel could be a product of different conformation of the standard proteins and the possible multimeric conformation of the *B. suis *urease.

*B. suis *urease activity was present between pH 6–8 but activity decreased sharply below pH 6. Recently the optimal pH of *B. abortus *urease was determined as 7.3 [21]. Similarly, the ureases from *Sthaphylococcus saprophyticus*, *S. salivarium*, *Ureaplasma urealyticum*, and *M. tuberculosis *have a neutral optimum pH between 7 and 7.5 [16,19,22,23]. In contrast, the ureases from *L. reuteri*, *L. fermentum*, *L. ruminis*, and *S. mitior *exhibit acidic optimal pH values of 2, 2, 3, and 4.5 respectively [19], while *H. pylori *and *K. aerogenes *have a slightly alkaline pH of 8.0 [8,24]. We assayed urease activity in Tris-HCl buffer at pH 8.0, as suggested by Dunn and co-workers [8]. Although Tris has been described as an inhibitor of urease, a simple control experiment comparing the activity in Tris-HCl and the synthetic buffer HEPES, showed no significant difference (results not shown).

In terms of temperature stability, ureases from different species of *Lactobacillus *have a very high optimal temperature between 55 to 65°C [19], and *M. tuberculosis *is stable between 22 to 60°C but is inactivated above 60°C [16]. *B. suis *urease was active between 10–45°C, and it was almost completely inactivated above 45°C. The optimal activity was observed between 28–35°C, temperatures that are close to the corporal temperature of humans and other animals who are hosts for this pathogen.

**Figure 4:**
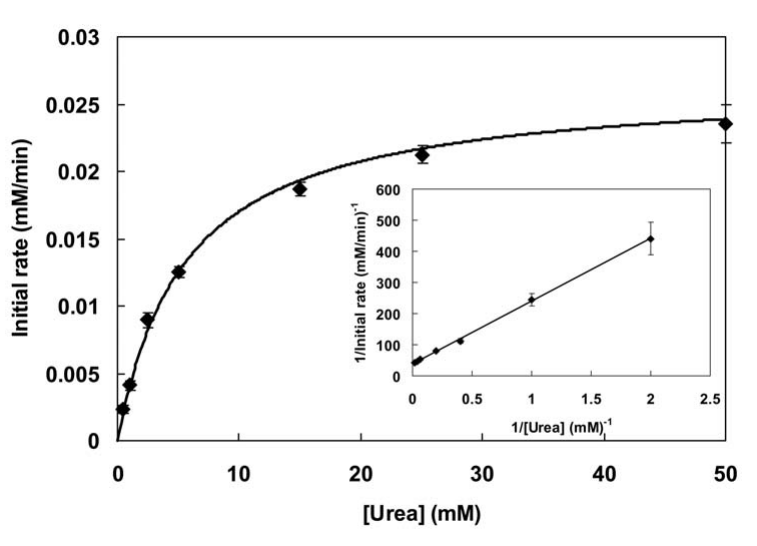
**Kinetics of the *B. suis *urease**. The graph shows the rectangular hyperbola obtained by nonlinear regression to the initial rate values, which yielded a K_m _(urea) of 5.60 ± 0.69 mM. The insert graph shows the Lineweaver-Burk plot. Initial rate values are the average of three determinations. Results shown are average ± standard deviation of triplicates of initial rate values for each substrate concentration, using the same enzyme preparation.

The kinetic study of *B. suis *urease showed a simple Michaelis-Menten-type kinetic behavior (Figure 4), in accordance with what has been described for ureases from other organisms [5]. The K_m _obtained by hyperbolic regression was 5.60 ± 0.69 mM, and a very similar value (5.24 mM) was obtained with the still widely used Lineweaver-Burk or double reciprocal plot (Figure 4). Hence, *B. suis *urease shows a higher affinity for its substrate than the enzymes from *B. abortus *(13 mM) [21], *P. mirabilis *(39 mM) or *Providencia stuartii *(12 mM) [25], but less affinity than *H. pylori *whose urease has one of the lowest *K*_*m *_values (0.3 mM) reported [8]. A limited correlation may exist between the value of this kinetic constant and the ecological niche of the host organism. For example, *H. pylori *inhibits the gastric mucosal lining, where low concentrations of urea are present (1.7 to 3.4 mM); such a low kinetic constant would allow this urease to function under close to saturation conditions despite the low substrate concentration. In contrast, microorganisms that are exposed to large amounts of urea such as are found in the urinary tract or in the soil typically possess ureases with higher K_m _values, such as 13, 60, and 40 to 130 mM observed for the enzymes from *P. mirabilis*, *Sporosarcina ureae*, and *Bacillus pasteurii*, respectively [5].

**Figure 5:**
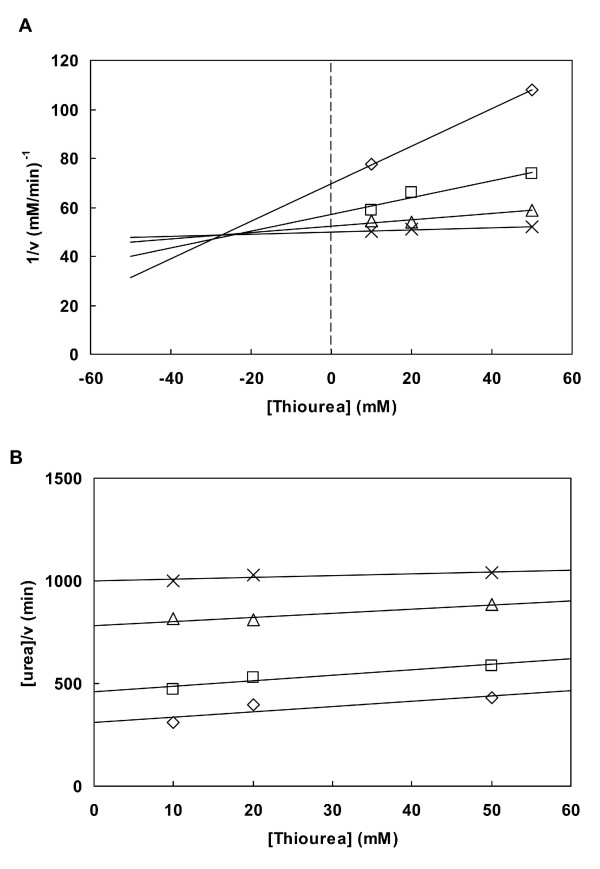
**Inhibition of *B. suis *urease by thiourea**. The concentrations of urea were 4 mM (□), 8 mM, 10 mM (Δ) and 20 mM (×). The combination of the two graphs, Dixon plot (A) and the parallel lines in graph (B) indicate a competitive inhibition, with a K_i _of 26.12 ± 2.30 mM determined from the intersection point of the lines in (A) [47]. Results are from a representative experiment using a single enzyme preparation.

Several compounds have been described as inhibitors of urease (for a review see [5,26]). These are potentially important to control urease related pathogenesis of bacterial infections and in enhancing the efficiency of urea fertilizers [5]. We tested the inhibitory effect of three of these compounds, hydroxyurea and thiourea, substrate analogues, and AHA. Both hydroxyurea and thiourea show a competitive type of inhibition (Figure 5), with K_i _of 1.04 ± 0.31 mM for hydroxyurea and 26.12 ± 2.30 mM for thiourea. These results agree well with what is described for ureases from other bacteria: the enzyme from *K. aerogenes *is inhibited by thiourea with a K_i _> 25 mM, and hydroxyurea is a competitive inhibitor of the *Brevibacterium ammoniagenes *urease with a K_i _of 0.23 mM [26]. For AHA, we used a similar approach and obtained a competitive inhibition with K_i _of 0.77 ± 0.06 mM. AHA has been described as a potent inhibitor of ureases from bacteria and other microorganisms [5] and the study of urease from jack bean and *K. aerogenes *revealed that AHA is a slow-binding competitive inhibitor with a very low K_i_, 2.6 – 4 μM [26,27]. Our results confirm that AHA is an inhibitor of *B. suis *urease, with a lower K_i _than thiourea and hydroxyurea. To test AHA as a slow binding inhibitor is beyond the scope of this study.

Due to the low protein concentration of the low molecular weight bands (Figure 2), it was not possible to sequence them. Since the active urease apoenzyme consists of UreA, UreB and UreC, it is likely that the smaller bands correspond to UreA and UreB. In the case of 66 kDa protein, we obtained the amino acid sequence MPARISRATYAQM; this sequence matched with *B. suis *UreC1 subunit (BR0270).

During the last years, several immunoreactive *Brucella *proteins have been identified and evaluated against animal and human serum samples [28-30]. It is well known that urease is the most abundant protein of *H. pylori *and as a result it serves as a significant immunogen and is useful in diagnostic tests. Patients with active gastritis due to *H. pylori *show significantly elevated immunoglobulin G and A sera titers to the urease [5]. In the current study, *B. suis *urease was tested for reactivity with human sera from patients with acute and chronic brucellosis by western blot analysis. The IgG antibodies in sera from patients with acute and chronic brucellosis reacted against the large subunit while the lower molecular weight subunits were not recognized. It is possible that the smaller subunits (UreA and UreB) did not react because their concentration was too low or that they are not immunogenic. As expected, healthy individuals were seronegative for reactivity to the UreC1 (data not shown). This is somewhat analogous to the serum reactivity of patients recovering from *Y. entercolitica *infections in which the UreB subunit is recognized [11].

Although the sequenced genomes of the *Brucella *spp. have two urease operons (*ure*-1 and *ure*-2), it appears that only one is responsible for encoding an active urease. In the case of *B. abortus*, Tn mutagenesis produced mutations of *ure-*1, but not *ure-2*, resulting in decreased urease activity [31]. Similarly, in the case of *B. suis*, the disruption of the *ure*C2 or *ure*B2, genes does not affect measurable urease activity in *B. suis *[16]. Thus, it is perhaps not unexpected that the urease purified in this study was identified as that encoded by *ure*C1 based on N terminal amino acid sequencing. Moreover, the serum from humans diagnosed with brucellosis also reacted with UreC1 and supports the mutational studies that only the *ure*-1 operon is used to produce urease during a *Brucella *infection. It is also noteworthy that the serum from patients infected with *Y. enterocolitica *also shows reactivity to the UreB subunit and is a candidate antigen involved in the induction of reactive arthritis in the same patients [32]. This ReA syndrome is also a typical side effect of a significant proportion of patients who have been infected with *Brucella *[33,34] or with a number of other pathogens acquired through the gastro-intestinal tract [35]. The question remains: why are the bacterial ureases so immunodominant and are they responsible, at least in part, for the induction of ReA as suggested by the arthritogenic peptide theory [36].

PSI-BLAST comparisons showed that UreC1 from *B. suis *has 99% of similarity with either the putative UreC1 from *B. melitensis *or UreC1 from *B. abortus *(encoded by BMEI0647 and BruAb1_0296 genes, respectively). Also, high similarity values were obtained by comparison with the alpha subunits from *M. loti *(81%), *R. leguminosarum *(78%), *S. meliloti *(79%) and *A. tumefaciens *(78%). In contrast, the comparison with UreC2 from *B. suis, B. melitensis *and *B. abortus *(BR1358, BMEI1652, and Bru Ab1_1355 genes, respectively), yielded lower values (around 55%, in each case). To find the relationships of the multiple urease clusters of *B. suis*, we carried out a phylogenetic and structure analysis with ureases from bacterial species with unique or multiple clusters.

**Figure 6:**
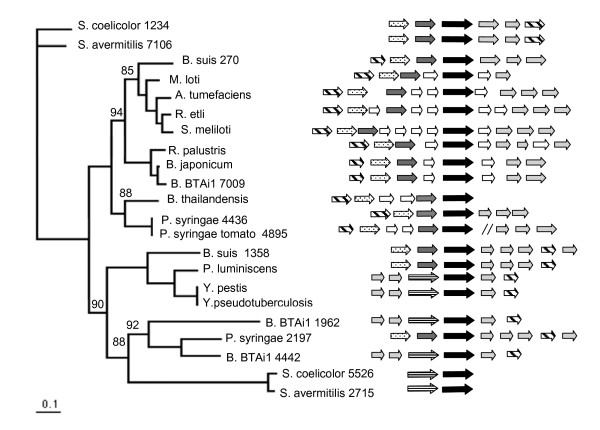
**Phylogenetic and cluster structure analysis**. Left. Phylogenetic tree inferred for UreC proteins. The tree topology and branch lengths were obtained with Maximum Likelihood method (as implemented in PhyML) with the JTT matrix. 100 bootstrap replicates were performed and the values, other than 100, are shown. *S. coelicolor *(SCO1234) was taken as outgroup. Right. Genetic structure of the clusters in each of the versions and species analyzed in the phylogenetic tree. Structures were obtained mainly from the MBGD database (see Methods). Boxes with arrows represent genes with relative direction of transcription. Fill-in code: dotted, *ureA*; dark gray, *ureB*; horizontal stripped, fusion of *ureA *and *ureB *genes; black, *ureC*; diagonal stripped, *ureD*; light gray, accessory genes *ureE, ureF *and *ureG*. When only two appear, lacking one is *ureE*. In *Bradyrhizobium *BTAi1, in the cluster of gene 1962, *ureH *appears instead of *ureD*. A fourth accessory gene, an urea transporter, appears in *B. suis *cluster 2 and *Y. pseudotuberculosis *near to *ureD*. White; hypothetical or unrelated genes. Slashes for *P. syringae *tomato denote that these genes are not contiguous.

The phylogenetic tree deduced for *B. suis *UreC1 (BR0270, denoted as *B. suis *270) grouped it within the *Rhizobiales *order (Figure 6, left). The closely related clades included the typical UreC from other alpha (not shown), beta (*Burkholderia thailandensis*), and gamma (*P. syringae and P. syringae *tomato) proteobacterial species. However, in the other main clade, the UreC2 (BR1358, denoted as *B. suis *1358) was grouped with *Yersinia *and *Photorhabdus *species, both from the gamma proteobacteria subdivision [bootstrap value = 100]. The unexpected phylogenetic relationship of cluster 2 possibly reflects a horizontal gene transfer event, because both *Brucella *and *Yersinia *share the niche (the mammalian cell), are classified as facultative intracellular pathogens and can replicate inside macrophages [1,37]. In a closely related clade appeared two UreC from *Bradyrhizobium *BTAi1 (denoted as 1962 and 4442) and one from *Pseudomonas syringae *strain B728a (PSYR2197). A similar explanation of horizontal gene transfer could be applied, because these species are common inhabitants of the soil and the plant rhizosphere. Also in this group were the UreC subunits of the distant Actinobacteria species *Streptomyces coelicolor *and *S. avermitilis*.

Interestingly, the main group relationships observed in the phylogenetic tree for UreC were also revealed in the structures of the urease clusters. In the alpha proteobacteria subdivision (including *Silicibacter pomeroyi*, *Rhodobacter sphaeroides*, *Rhodopseudomonas palustris*, not shown), the Rhizobiales and *B. suis*, the cluster 1 structure has the order *ureDABCEFG *(Figure 6, right). A slight variation, with the structure *ureDABCFG*, is present in *B. japonicum*, in one of the clusters of *Bradyrhizobium *strain BTAi1 (denoted as 7009), in some gamma proteobacteria such as *Pseudomonas aeruginosa *(not shown) and in one of the two clusters of *P. syringae *strain B728a; in two last species, the *ureFG *genes are located several genes downstream. A second main structure, with the order *ureABCEFGD*, was shared by *B. suis *cluster 2 and the *Yersinia pestis, Y. pseudotuberculosis *and *Photorabdus luminiscens *clusters and with one of the clusters of *Streptomyces coelicolor *and *S. avermitilis *(*Actinobacteria*). Lastly, a third structure was found with the order *ureEF(AB)CGD*. This structure was present in two of the clusters of *Bradyrhizobium *strain BTAi1 (genes 1962 and 4442) and in one cluster of *P. syringae *strain B728a. In this case (AB) symbolizes the fusion of *ureA *and *ureB *genes. In *Streptomyces coelicolor *and *S. avermitilis *the urease clusters consist of only *ure(AB)C *genes. The conservation of the cluster and the coherence with the phylogenetic tree among the analyzed species suggests a coordinated evolution of genes and clusters and, perhaps, a relationship between origin and function.

## Conclusion

We have for the first time purified and characterized an active urease from *B. suis*. The enzyme was characterized at the kinetics, immnunological and phylogenetic levels. Our results confirm that the active urease of *B. suis *is product of *ure*-1 operon.

## Methods

### Bacterial strains and growth

*B. suis *1330 (ATCC 23444) was kindly donated by Central Veterinary Laboratory (New Haw, Weybridge, United Kingdom). The strain was grown on Trypticase soy agar (Difco) supplemented with 0.5% (w/v) yeast extract (Difco) (TSA-Y) at 37°C for 48 h.

### Preparation of cell extract

The crude extract was obtained by breaking the cells with glass beads treatment as previously described [38]. The extract was concentrated using an Amicon Ultra, 5 K (Millipore Corporation), aliquoted and stored at -20°C.

### Enzyme purification

The sample was applied onto a XK26 column packed with High Performance Q Sepharose (20 ml) equilibrated with 10 mM imidazole buffer (pH 7.0). A gradient of 0 to 1 M NaCl was applied at a flow rate of 120 ml/h, and 5 ml fractions were collected and assayed for urease activity. Fractions with urease activity were pooled and saturated with 1.5 M ammonium sulfate. The sample was then loaded to a XK26 column packed with Source PHE (20 ml) previously equilibrated with 100 mM sodium phosphate and 1.5 M ammonium sulfate (pH 7.0). The column was eluted with a descending gradient from 1.5 to 0 M ammonium sulfate at a flow rate of 30 ml/h. All purification steps were performed at 4°C using a fast protein liquid chromatography (AKTA FPLC) system (Amersham Pharmacia Biotech, Uppsala, Sweden).

### Enzyme assays

Three different assays were used: (a) *Phenol red urease test*. Qualitative urease test were performed during enzyme purification. Urease activity was monitored by observing the color change of a phenol red pH indicator from yellow to red, which results from a pH increase due to urea hydrolysis. Extract fractions (10 μl) were mixed with 100 μl of assay phosphate buffer pH 7.0 containing 1 mM free-acid form of phenol red and 100 mM urea and incubated at 28°C. (b) *Spectrophotometric urease assay using phenol red*. Urease activity was quantitated spectrophotometrically as previously described [20,39]. (c) *Quantitative urease assay*. Quantitative measurements of urease activity were determined spectrophotometrically using a coupled assay with glutamate dehydrogenase, as previously described [31,40]. One unit of urease activity was defined as the amount of enzyme that hydrolyzes 1 μmol of urea per min. Specific activities were calculated as units of urease per mg of protein in the extract.

### Protein determination

Protein concentrations were determined by the bicinchoninic acid assay (Pierce, Rockford, Ill) or using Coomassie Brilliant Blue G in a Bradford-modified assay, as decribed [41].

### Native and denaturing polyacrylamide gel electrophoresis

Native polyacrylamide gel electrophoresis was performed using polyacrylamide gradient from 5 to 10% and detection of urease on the native gel was performed as described by Mobley *et al*. [5]. A kit for molecular weights 14,000–500,000 Da for non-denaturing PAGE was used to estimate the native urease molecular weight (Sigma-Aldrich, St. Louis, Miss.). Denaturing gel electrophoresis (SDS-PAGE) was performed on 15% polyacrylamide gels as described by Laemmli [42] and used to monitor purification and estimate the molecular weight of the protein. Gels were stained with Coomassie Blue G250. A broad molecular marker set (200,000 to 6,500 kDa, Biorad Lab, Inc) was used as a standard.

### Amino acid sequencing

For amino-terminal sequence analysis, the UreC1 putative band resolved by SDS-PAGE was electrotransferred to a polyvinylidene difluoride membrane [43] and the N-terminal sequence was determined by automated Edman degradation (Synthesis and Sequencing Facility at Johns Hopkins University). The amino-acid sequence obtained was compared with available sequences at the website of the National Center of Biotechnology Information [44] using the Psi-BLAST program [45].

### Molecular weight determination

The relative molecular weight of urease was estimated by FPLC gel filtration and SDS-PAGE. Gel filtration was executed on a Superose 12 (10/300) column (Amersham Pharmacia Biotech, Uppsala, Sweden) calibrated with the following standard markers: thryroglobulin (670 kDa), gamma globulin (158 kDa), ovalbumin (44 kDa), myoglobin (17 kDa) and vitamin B12 (1.3 kDa) (Bio-Rad). Equilibrium and elution (0.5 ml/min) were performed with 50 mM phosphate buffer pH 7.0 containing 150 mM NaCl.

### Determination of pI

The isoelectric point was determined by chromatofocusing on a MonoP FPLC column equilibrated with 25 mM imidazole, pH 7.4 using a pH gradient from 7.0 to 4.0 (Polybuffer 74, Amersham Pharmacia Biotech, Uppsala, Sweden).

### Influence of temperature and pH on urease activity

Activity of urease was examined in 15 mM sodium acetate at pH values ranging from 2, 3 and 4; in 20 mM sodium phosphate for pH 5, 6, 7, and 8.2; and in 25 mM glycine-25 mM sodium hydroxide-25 mM sodium chloride for pH 9, 10, and 10.6. Urease samples were incubated for 5 min at 28°C in the different pH buffers. After incubation, urea was added to a final concentration of 5 mM and urease activity was determined using the quantitative coupled assay described above. The influence of temperature on urease activity was determined by equilibration of urease samples at various temperatures between 5 to 70°C for 5 min in 30 mM Tris-HCl buffer pH 7.0, and urease activities were determined by the spectrophotometric urease assay using phenol red described above.

### Determination of kinetic parameters

Kinetics studies were performed using the same coupled assay described for specific activity determination, described above. K_m _for urea was determined within a concentration range of 0.25 to 25 mM. The reactions were followed for 5 min and initial rates were calculated from the linear portion of the curve. Data was fitted to irreversible single substrate Michaelis-Menten models (rectangular hyperbola) by non-weighted non-linear regression using the program Hyperfit [46].

### Inhibition studies

Thiourea, hydroxyurea and acetohydroxamic acid (AHA) were tested as inhibitors of *B. suis *urease. The inhibition studies were performed as indicated by Cornish-Bowden [47]. Thiourea inhibition was determined using 4, 8, 15 and 20 mM of urea and, for each of these concentrations, varying the inhibitor from 10 to 50 mM. Hydroxyurea was used in a concentration range of 0.5 to 20 mM for the same concentrations of substrate described before. AHA was studied using the same substrate concentration range and 0.1, 0.5 and 1 mM of inhibitor. In all cases, the urease activity was determined as described before, and appropriate controls were made to ensure that none of the inhibitors tested inhibited the auxiliary enzyme, glutamate dehydrogenase.

### Human sera samples

Sera from 9 patients with brucellosis at different stages of the disease were used. The samples were selected according to the following criteria: 5 patients had acute brucellosis as determined by the clinical symptoms and an agglutination titer equal to or higher than 1:80, and with positive 2-mercaptoethanol (2-ME) agglutination. All 5 blood cultures of acute patients were positive for *B. melitensis*. The other 4 patients were considered to have chronic brucellosis as determined by a history of persisting symptoms or relapses and persistent agglutination titers (≥ 1:20), over the course of more than one year. Sera from 21 volunteers, without antecedents of brucellosis, and negative to standard and 2-ME agglutination tests were included.

### Western blot

Immunoblotting was carried out by the method of Towbin and co-workers [48]. After transfer of the proteins from SDS-PAGE, the membrane was blocked with 2% albumin and cut in 4-mm strips. Samples of human sera were diluted 1:20 in Tris-saline buffer (TSB), and then added to each strip of membrane and incubated overnight at 4°C. Following incubation the strips were washed and 0.5 ml of a 1:500 dilution of horseradish peroxidase labeled anti-immunoglobulin G (Cappel, USA) in TBS was added. After incubation for 1 h at 37°C, the conjugate solution was discarded and strips were washed with TBS. Finally, the strips were developed by the addition of 10 ml of TBS containing 5 mg of diamino-benzidine (Gibco BLR, USA) and 0.1% hydrogen peroxide. Prestained molecular mass markers (Gibco BRL, USA) were used as standards.

### Phylogenetic and gene cluster analyses

The phylogenetic tree for UreC was performed with the alignment of the corresponding amino acid sequences with ClustalW [49] and applying the algorithm of Neighbor-Joining [50] with a bootstrap of 100 replicates, as implemented in the Phylip package v3.2 [51]. Additionally, a tree applying the Maximum likelihood method, as implemented in PhyML [52] was obtained with JTT distances. Identical grouping in the trees was obtained with both methods. *S. coelicolor *was chosen as outgroup and the trees were graphed with TreeView v1.6.6. [53]. For gene context analysis, the cluster structures were defined using the information from MBGD database [7] for each published genome, and the Rhizobase [54] in the case of *Bradyrhizobium *strain BTAi1. The genomes analyzed and accession numbers for UreC sequences are as follows: *Bradyrhizobium japonicum *USDA110 (Q89UG0), *Agrobacterium tumefaciens *C58 (Q8UCT2), *Rhizobium etli *CFN42 (YP470796), *Sinorhizobium meliloti *1021 (AAB30138), *Brucella suis *1330 (Q8G2P9, BR270), *B. suis *1330 (Q8FZW3, BR1358), *Mesorhizobium loti *MAFF303099 (NP105696), *Rhodopseudomonas palustris *CGA009 (Q6N3N3), *Photorhabdus luminescens *TT01 (NP929433), *Yersinia pestis *KIM (AAM84814), *Yersinia pseudotuberculosis *IP32953 (CAH22180), *Pseudomonas syringae *pv tomato DC3000 (AA058324), *P. syringae *B728a (YP237504, PSYR4436), *P. syringae *B728a (YP235278, PSYR2197), *Burkholderia thailandensis *(YP442042), *Streptomyces coelicolor *A3(2) (NP629660, SCO5526), *S. coelicolor *A3(2) (NP625522, SCO1234), *S. avermitilis *(Q82JN9, SAV2715), *S. avermitilis *(Q826R9, SAV7106). Not shown in the gene context figure but mentioned in the text are: *Silicibacter pomeroyi *DSS-3, *Rhodobacter sphaeroides *241 and *Pseudomonas aeruginosa *PAO1. In the case of *Bradyrhizobium *strain BTAi1, the data was obtained from the finished genomic sequence (accession number NC 009485, [6]). The *ureC *genes begin at following positions: Bbta_1962: 2026259, Bbta_4442: 4653784, Bbta_7009: 7345069. The predictions for urea transporters (for *B. suis *BR1358 gene in cluster 2 and *Y. pseudotuberculosis *YPTB2937) were obtained from the KEGG [55] and MBGD [7] databases.

## Abbreviations

Acetohydroxamic acid: AHA; HEPES: 4-(2-hydroxyethyl)-1-piperazineethanesulfonic acid; 2-mercaptoethanol: 2-ME; sodium dodecylsulfate – polyacrylamide gel electrophoresis: SDS-PAGE.

## Authors' contributions

AC-R carried out the laboratory work and wrote the draft of the manuscript. JQ-L and EA-C carried out part of the purification and biochemical characterization. AMM performed the determination of kinetic parameters and inhibition studies. HP performed the phylogenetic and gene cluster analyses. AL-M, NS and SMB conceived of the study and participated in its design, coordination and helped to write the final manuscript. All authors read and approved the manuscript.
